# *Campylobacter concisus* pathotypes induce distinct global responses in intestinal epithelial cells

**DOI:** 10.1038/srep34288

**Published:** 2016-09-28

**Authors:** Nandan P. Deshpande, Marc R. Wilkins, Natalia Castaño-Rodríguez, Emily Bainbridge, Nidhi Sodhi, Stephen M. Riordan, Hazel M. Mitchell, Nadeem O. Kaakoush

**Affiliations:** 1 Systems Biology Initiative, School of Biotechnology and Biomolecular Sciences, UNSW Australia, Sydney 2052, NSW, Australia; 2 School of Biotechnology and Biomolecular Sciences, UNSW Australia, Sydney 2052, NSW, Australia; 3 Ramaciotti Centre for Gene Function Analysis, UNSW Australia, Sydney 2052, NSW, Australia; 4 Gastrointestinal and Liver Unit, The Prince of Wales Hospital, Randwick, NSW 2031, Australia; 5 School of Medical Sciences, UNSW Australia, Sydney 2052, NSW, Australia

## Abstract

The epithelial response to the opportunistic pathogen *Campylobacter concisus* is poorly characterised. Here, we assessed the intestinal epithelial responses to two *C. concisus* strains with different virulence characteristics in Caco-2 cells using RNAseq, and validated a subset of the response using qPCR arrays. *C. concisus* strains induced distinct response patterns from intestinal epithelial cells, with the toxigenic strain inducing a significantly more amplified response. A range of cellular functions were significantly regulated in a strain-specific manner, including epithelial-to-mesenchymal transition (NOTCH and Hedgehog), cytoskeletal remodeling, tight junctions, inflammatory responses and autophagy. Pattern recognition receptors were regulated, including *TLR3* and *IFI16*, suggesting that nucleic acid sensing was important for epithelial recognition of *C. concisus*. *C. concisus* zonula occludens toxin (ZOT) was expressed and purified, and the epithelial response to the toxin was analysed using RNAseq. ZOT upregulated *PAR2* expression, as well as processes related to tight junctions and cytoskeletal remodeling. *C. concisus* ZOT also induced upregulation of *TLR3*, pro-inflammatory cytokines *IL6*, *IL8* and chemokine *CXCL16*, as well as the executioner caspase *CASP7*. Here, we characterise distinct global epithelial responses to *C. concisus* strains, and the virulence factor ZOT, and provide novel information on mechanisms by which this bacterium may affect the host.

*Campylobacter concisus* is a member of the human oral microbiota^1^,^2^, that has also been detected in the oesophagus^3^ and lower intestinal tract^1^ of healthy humans. *C. concisus* has also been associated with a range of human diseases such as Barrett’s oesophagus^4^, gastroenteritis^5^,^6^,^7^ and inflammatory bowel diseases^8^,^9^.

A number of possible explanations have been suggested for the presence of *C. concisus* in both healthy and diseased individuals. For example, *C. concisus* may act as an opportunistic pathogen whose growth and pathogenesis is modulated by host factors such as exposure to inflammation, acid, or bile^4^,^10^ or by the host microbiota^11^. Further, *C. concisus* strains have now been classified into commensals and at least two different pathotypes [adherent toxigenic *C. concisus* (AToCC) and adherent invasive *C. concisus* (AICC)] based on their virulence mechanisms^12^. One such virulence mechanism pertinent to AToCC strains is the presence of a zonula occludens toxin (ZOT)^13^, a toxin that was first identified in *Vibrio* species to target tight junctions of epithelial cells^14^. A further virulence mechanism present in AICC strains is the ability to survive intracellularly within epithelial cells in a process that involves autophagy^15^. In addition to these differences, the strains belonging to these pathotypes have been shown using comparative genomics to be highly genetically diverse^16^.

However, despite an improvement in our understanding of the bacterium’s virulence mechanisms, the epithelial response to *C. concisus* remains poorly understood. What is clear from our current knowledge of *C. concisus*-epithelial interaction, is that in addition to the role of autophagy and tight junctions^15^,^17^, host cells produce a range of pro-inflammatory cytokines in response to infection^10^,^18^. Thus, to gain a better understanding of the interaction between *C. concisus* and the host at the epithelial interface, this study comprehensively assessed the response of the intestinal epithelial cell-line Caco-2 to AToCC strain BAA-1457 and AICC strain UNSWCD with RNA-seq. The response of Caco-2 cells to purified ZOT from *C. concisus* BAA-1457 was also determined using the same approach.

## Results

### Comparative analysis of epithelial response to *Campylobacter concisus* pathotypes

**Figure 1:**
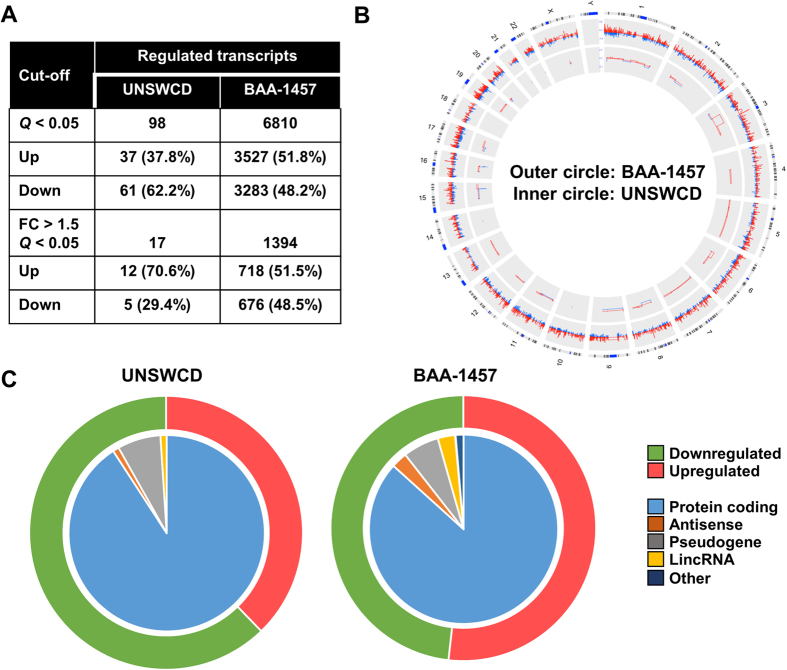
Transcriptional regulation in intestinal epithelial Caco-2 cells infected with *Campylobacter concisus*. (**A**) Number of significantly differentially expressed transcripts following infection with *C. concisus* BAA-1457 or UNSWCD. (**B**) Comparison of the responses of Caco-2 cells to *C. concisus* BAA-1457 (outer circle) and UNSWCD (inner circle). Transcripts were mapped according to location on human chromosomes. Responses were visualised using circular Circos plot, with transcripts increased in expression in red and those decreased in blue. (**C**) Numbers of upregulated (red) and downregulated (green) transcripts visualised in the outer circles, and types of regulated transcripts visualised independently in the inner circles.

The response of Caco-2 intestinal epithelial cells (IECs) to two *C. concisus* strains with different virulence mechanisms was examined using RNA-seq. Global gene expression indicated that IECs responded in a distinct manner to each of the AICC (UNSWCD) and AToCC (BAA-1457) strains, with the response to the AToCC strain being markedly larger (Fig. 1A,B). A similar number of transcripts were upregulated and downregulated following infection with BAA-1457, while a higher number of transcripts were downregulated than upregulated following infection with UNSWCD (Fig. 1C). The majority of differentially expressed transcripts were protein coding, however, a range of antisense and long non-coding transcripts were also identified to be differentially expressed (Fig. 1C).

### Epithelial response to infection with adherent invasive *Campylobacter concisus*

Ninety-eight transcripts were found to be differentially expressed (*Q* < 0.05) following infection of IECs with the AICC strain UNSWCD (Fig. 1A, Supplementary Data 1). When the threshold was increased (*Q* < 0.1), the number of transcripts only increased to 156 (Supplementary Data 1). This epithelial response was weak when compared to the response of THP-1 derived macrophages^19^, which differentially expressed 8,343 transcripts at greater than 1.5-fold regulation (*Q* < 0.05) following infection with *C. concisus* UNSWCD.

Infection with *C. concisus* UNSWCD induced an oxidative stress response in IECs, with a range of metallothionein-encoding transcripts found to be upregulated, in addition to nitric oxide synthase 2 (*NOS2*) and glutathione peroxidase 1 (*GPX1*) (Supplementary Data 1). Further, transcripts involved in xenobiotic metabolism (*CYP1A1* and *CYP1B1*) and fluid transport (*NPPB* and *VIPR1*) were also upregulated.

Transcripts involved in the inflammatory response were also differentially expressed (upregulated) following infection with *C. concisus* UNSWCD. *CCL2* and *BIRC3*, as well as *IKBKE*, which is involved in the non-canonical activation of NF-κB, were upregulated (Supplementary Data 1). There also appeared to be an impact on the tight junctions of IECs, with a range of transcripts showing significant changes in expression in both directions (*CLDN2*, *PMP22*, *CLDN1*, and *TJP1*). One transcript (*ATG9B*) involved in autophagy was significantly decreased in expression following infection. Given the importance of autophagy in the intracellular survival of *C. concisus*, we confirmed the repression of this process following infection with another AICC strain, UNSW3 (Table 1). Further, a range of transcripts (*SQLE*, *LDLR*, *VLDLR*, *HMGCS1*, and *INSIG1*) involved in cholesterol synthesis, metabolism and transport were significantly decreased in expression following infection with *C. concisus* UNSWCD.

### Epithelial response to infection with adherent toxigenic *Campylobacter concisus*

**Figure 2:**
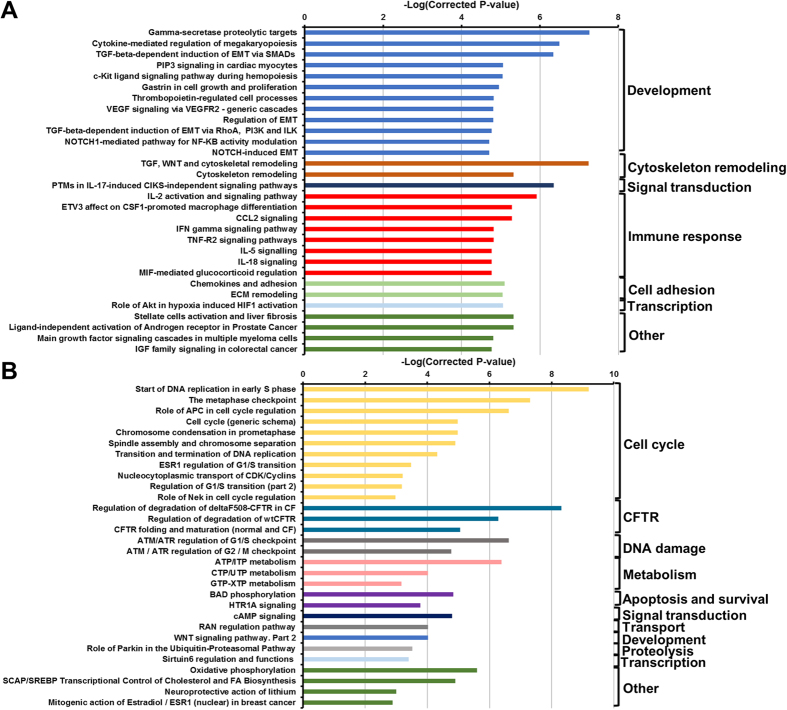
Pathways significantly regulated in intestinal epithelial Caco-2 cells infected with *Campylobacter concisus* BAA-1457. Top 30 upregulated (**A**) and downregulated (**B**) pathways were selected. Pathways were identified using MetaCore and inverse of log(FDR corrected P-value) was plotted. Full list of top 50 upregulated and downregulated pathways can be found in Supplementary Data 2.

**Figure 3:**
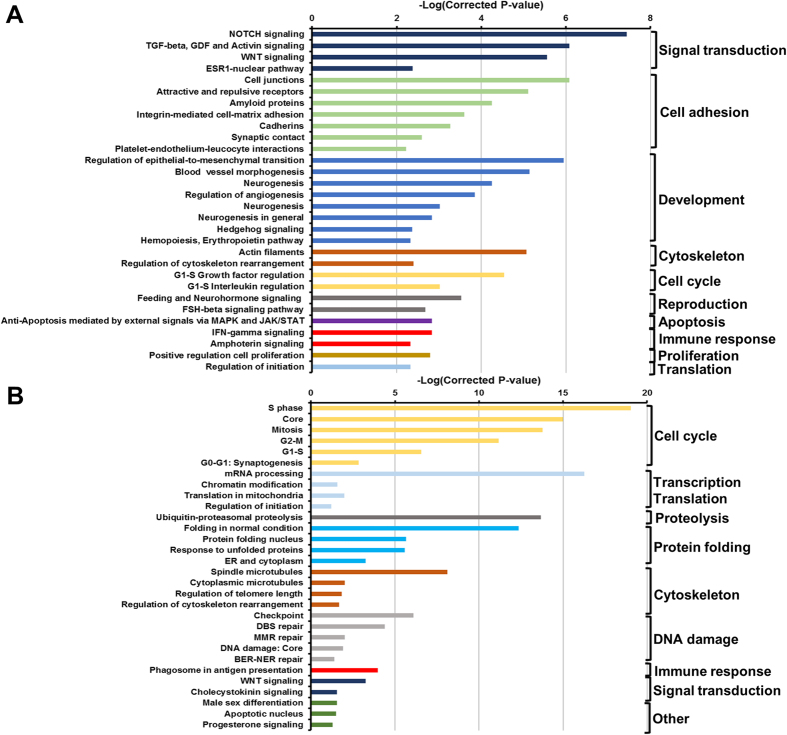
Processes significantly regulated in intestinal epithelial Caco-2 cells infected with *Campylobacter concisus* BAA-1457. Top 30 upregulated (**A**) and downregulated (**B**) processes were selected. Processes were identified using MetaCore and inverse of log(FDR corrected P-value) was plotted. Full list of top 50 upregulated and downregulated processes can be found in Supplementary Data 2.

Infection of IECs with the AToCC strain BAA-1457 significantly affected the expression of 6810 transcripts, of which 1394 were differentially expressed at levels greater than 1.5-fold or less than 0.75-fold (Fig. 1A, Supplementary Data 1). A range of pathways and processes were found to be significantly enriched in each of the upregulated and downregulated datasets (Figs 2A,B and 3A,B, Supplementary Data 2). Pathways and processes related to epithelial-to-mesenchymal transition (EMT) were enriched in the upregulated dataset, in particular NOTCH signaling and Hedgehog signaling (Figs 2A and 3A). Processes related to cytoskeletal remodeling and cellular junctions, as well as inflammatory responses (IL-2, IL-5, IL-18, CCL2, TNF, IFN, and TGF-β signaling) were also upregulated following infection with *C. concisus* BAA-1457 (Figs 2A and 3A). In contrast, cystic fibrosis transmembrane conductance regulator (*CFTR*)-associated pathways, cell cycle processes (S-phase and others), and DNA damage processes were all downregulated following infection (Figs 2B and 3B).

In order to identify molecules that could be involved in epithelial recognition of *C. concisus* BAA-1457, the expression of pattern recognition receptors (PRRs) and related transcripts were examined (Table 2). The expression of PRRs involved in sensing nucleic acids (*TLR3* and *IFI16*) and the NOD-like receptor NLRP9 increased following infection. The expression of the cytokines IL-18, IL-34 and chemokine CCL28 also increased following infection (Table 2).

The differential expression of genes involved in autophagy was also identified in IECs infected with *C. concisus* BAA-1457 (Supplementary Data 1). Importantly, the expression of *MTOR*, a master inhibitor of autophagy, was decreased (0.78-fold) following infection with *C. concisus* BAA-1457. To further validate the modulation of transcripts within the autophagy pathway, we determined the expression of 84 autophagy-related genes and five housekeeping genes following infection with *C. concisus* BAA-1457 using an autophagy-specific qPCR array. The average triplicate Ct values arising from infected and non-infected cells were normalised against the housekeeping genes *B2M* and *HPRT1*. These housekeeping genes were chosen as they produced the lowest difference in geometric mean between the control and test group. In total, four genes were differentially expressed at statistically significant levels: *ATG4A*, *EIG4G1*, *MAP1LC3B* and *MTOR* (Table 1). The expression of *ATG4A*, *EIG4G1*, and *MTOR* were all decreased in both datasets, while the expression of *MAP1LC3B*, which was not found to be modulated in the RNAseq data, was found to be significantly increased in the qPCR data (Table 1).

### Epithelial response to exposure to *Campylobacter concisus* zonula occludens toxin

**Figure 4:**
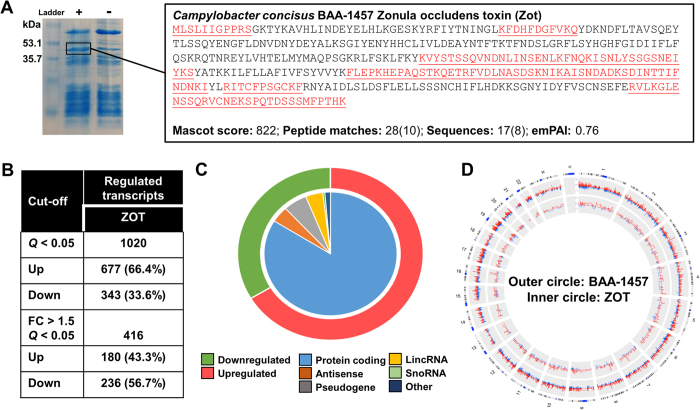
Transcriptional regulation in intestinal epithelial Caco-2 cells exposed to zonula occludens toxin. (**A**) *In vitro* expression of ZOT and confirmation of protein identity using mass spectrometry. (**B**) Number of significantly regulated transcripts following exposure of Caco-2 cells to ZOT. (**C**) Numbers of upregulated (red) and downregulated (green) transcripts visualised in the outer circle, and types of regulated transcripts visualised independently in the inner circle. (**D**) Comparison of the responses of Caco-2 cells to *C. concisus* BAA-1457 (outer circle) and ZOT (inner circle). Transcripts were mapped according to location on human chromosomes. Responses were visualised using circular Circos plot, with transcripts increased in expression in red and those decreased in blue.

Zonula occludens toxin from *C. concisus* BAA-1457 was expressed, and its identity subsequently confirmed using mass spectrometry (Fig. 4A). Exposure of IECs to purified ZOT from *C. concisus* BAA-1457 significantly changed the expression of 1020 transcripts, of which 416 were regulated at levels greater that 1.5-fold or less than 0.75-fold (Fig. 4B, Supplementary Data 1). The majority (66.4%) of significantly differentially expressed transcripts were upregulated (Fig. 4B,C), and 83.9% of the total number of differentially expressed transcripts corresponded to protein coding transcripts (Fig. 4C). However, a substantial proportion of regulated transcripts were antisense or long non-coding RNAs (8.4%, Fig. 4C). Comparison of the IEC response to *C. concisus* BAA-1457 and purified ZOT suggested that the response to the toxin was more specific (Figs 1A and 4B), while the response to the strain was stronger (Fig. 4D). Of the 1020 transcripts differentially expressed by exposure to ZOT, 555 (54.4%) showed an overlap with the BAA-1457 response (Supplementary Data 1).

**Figure 5:**
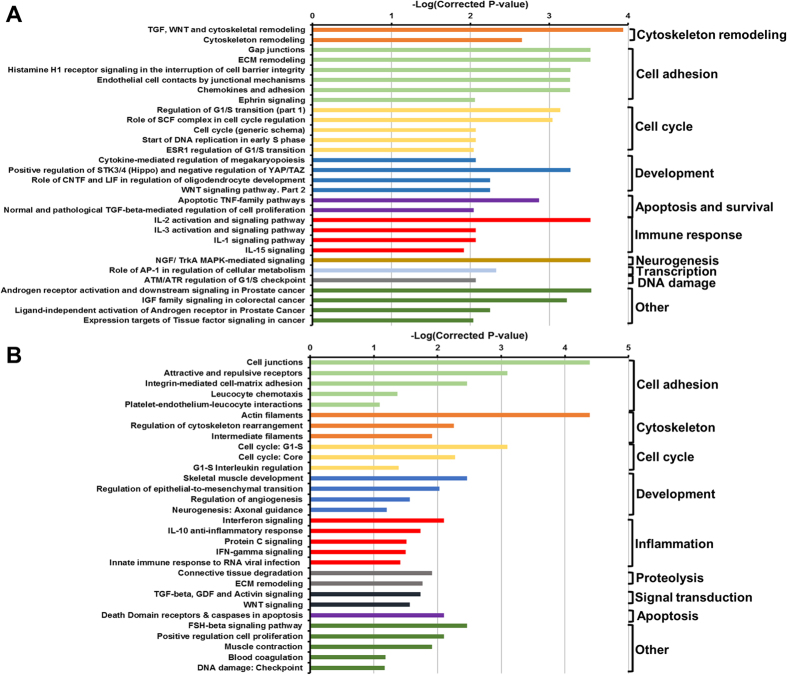
Pathways and processes significantly upregulated in intestinal epithelial Caco-2 cells exposed to zonula occludens toxin. Top 30 upregulated pathways (**A**) and processes (**B**) were selected. Pathways and processes were identified using MetaCore and inverse of log(FDR corrected P-value) was plotted. Full list of top 50 upregulated and downregulated pathways and processes can be found in Supplementary Data 2.

Exposure to ZOT significantly regulated a range of pathways and processes in IECs (Fig. 5A,B, Supplementary Data 2). Similar to the IEC response to *C. concisus* BAA-1457, processes related to EMT, cytoskeletal remodeling and cellular junctions were upregulated following exposure to ZOT (Fig. 5A,B). Importantly, processes related to cellular junctions and actin filaments were the most enriched within the upregulated dataset (Fig. 5B). Further, a number of inflammatory responses (IL-1, IL-2, IL-3, IL-5, IL-15, and TNF signaling) were also upregulated upon exposure to ZOT (Fig. 5A,B). While several pathways and processes were identified to be significantly downregulated following exposure to ZOT (Supplementary Data 2), the number of transcripts within the pathways identified to be regulated was very low when compared to the total number of transcripts in each of the pathways.

The differential expression of PRRs and related transcripts following exposure to ZOT were examined (Table 3). Interestingly, the expression of both *TLR3* and *NLRP2* were increased in IECs exposed to ZOT, as was found for IECs infected with *C. concisus* BAA-1457 (Tables 2 and 3). Further, IECs increased expression of *IL6*, *IL8*, and *CXCL16* following exposure to ZOT (Table 3), indicating a level of similarity between the IEC response to BAA-1457 and its toxin. However, in contrast to the response to *C. concisus* BAA-1457, exposure to ZOT did not affect genes involved in autophagy or DNA-sensing IFI16 (Supplementary Data 1), emphasising that these were responses specific to the *C. concisus* BAA-1457 cells. Of particular relevance to ZOT function, *PAR2* expression was significantly increased upon infection with *C. concisus* BAA-1457 (1.2-fold), and more so upon exposure to purified ZOT (1.6-fold).

### Validation of the intestinal epithelial responses to *Campylobacter concisus* strains

We validated in two cell-lines (Caco-2 and HT-29) the changes in expression of a number of genes (*MT1E*, *NPPB*, *NOTCH3*, *CYP26A1*, *HEY1* and *CYP1A1*) found to be differentially expressed using RNAseq following infection with *C. concisus* UNSWCD or BAA-1457 (Supplementary Data 3). Overall, there was good concordance between the RNAseq and qPCR data and between the two cell-lines, specifically in their metallothionein, natriuretic peptide, and cytochrome P450 responses. Further, *HEY1*, a gene involved in EMT, was consistently upregulated in both cell-lines following infection with strain BAA-1457.

A number of similarities were observed in the response to the potential commensal strain ATCC 51561 (*NPPB* and *CYP1A1*); however, a number of genes (e.g. *MT1E*, *NOTCH3*, *CYP26A1*, *HEY1*) found to be differentially expressed by infection with *C. concisus* UNSWCD or BAA-1457 were not modulated by this strain.

## Discussion

*Campylobacter concisus*, a commensal within the oral cavity, is an emergent opportunistic pathogen of the gastrointestinal tract, having been associated with Barrett’s oesophagus, gastroenteritis, and inflammatory bowel diseases^3^,^4^,^5^,^6^,^7^,^8^,^9^. A range of virulence mechanisms and factors have been identified in *C. concisus* strains^13^,^18^, which has led to the division of strains into at least two pathotypes, adherent-invasive (AICC) and adherent-toxigenic (AToCC) strains^12^. Recently, our group comprehensively characterised the response of macrophages to *C. concisus* strains using RNAseq, identifying DNA sensing as an important mechanism by which host immune cells recognise *C. concisus*^19^. However, despite an increase in our understanding of the virulence mechanisms and immune response to *C. concisus*, the epithelial response to the bacterium remains poorly understood.

Thus, the responses of IECs to either AICC or AToCC strains were determined using RNAseq. Large-scale differences in the responses of IECs to each of the pathotypes were observed, with the AToCC strain inducing a significantly larger response upon infection of IECs. We have previously found that *C. concisus* UNSWCD (AICC) can invade IECs and survive intracellularly, while *C. concisus* BAA-1457 (AToCC) can internalise into IECs but is cleared from the intracellular environment by autophagy^15^. These findings would suggest that AICC strains appear to avoid detection by IECs, and their ensuing protective response, allowing them to establish a niche intracellularly. However, despite the lack of a strong epithelial response to *C. concisus* UNSWCD, an AICC strain, a specific protective response was still observed. For example, the upregulation of *IKBKE* following infection with *C. concisus* UNSWCD would suggest non-canonical activation of NF-κB^20^, which supports our previous observation of NF-κB activation in IECs exposed to AICC strains^18^. Moreover, a strong oxidative stress response in the form of increased expression of metallothioneins and other antioxidant enzymes, as well as the upregulation of xenobiotic metabolism (*CYP1A1* and *CYP1B1*) were found following infection with *C. concisus* UNSWCD.

In contrast, *C. concisus* BAA-1457 (AToCC), which produces a zonula occludens toxin, does not avoid detection by IECs, but rather induced a strong epithelial response. This is further emphasised by the decreased expression of *MTOR* and *EIF4GI* following infection with *C. concisus* BAA-1457, both of which are inversely associated with activation of autophagy^21^. Further, IECs significantly increased expression of two nucleic acid sensing PRRs, *TLR3* and *IFI16*, following infection with *C. concisus* BAA-1457, which is suggestive of bacterial lysis within the host cell. Notably, increased expression of *IFI16* and the formation of the IFI16 inflammasome were observed in immune cells infected with *C. concisus* BAA-1457^19^, highlighting the importance of host DNA sensing in the recognition of certain *C. concisus* strains.

In addition to the above PRRs, we also observed a small increase in the IEC expression of *NLRP2* following both infection with *C. concisus* BAA-1457, as well as exposure to the purified ZOT. Interestingly, NLRP2 has been found to mediate the production of β-defensins in gingival epithelial cells infected with *Fusobacterium nucleatum*^22^.

Infection with *C. concisus* BAA-1457 significantly modulated several pathways associated with CFTR, which was not observed in IECs exposed to *C. concisus* ZOT. Given that *Vibrio cholerae* targets CFTR through the cholera toxin present on the CTX prophage, which also comprises a zonula occludens toxin^23^,^24^, it is conceivable that *C. concisus* BAA-1457 may have acquired through horizontal gene transfer additional toxins capable of targeting CFTR at the time of acquiring ZOT. Analysis of the *C. concisus* genome suggests ZOT is present on an incomplete prophage^25^, thus, proteins within this prophage would serve as possible candidates.

*Campylobacter concisus* BAA-1457 was also found to upregulate a range of pathways and processes associated with EMT, as well as IGF family signaling in colorectal cancer. Further, the strain isolated from a healthy individual (ATCC 51561) did not appear to upregulate EMT. Notably, studies have found that *Campylobacter* species are among a polymicrobial signature of Gram-negative bacteria (including *Fusobacterium*) that are enriched in colorectal cancer patient samples^26^,^27^, suggesting that the presence of some types of *C. concisus* strains in the gut may have the potential to promote functions associated with carcinogenesis. Moreover, a similar polymicrobial signature comprising *Campylobacter* and *Fusobacterium* species is enriched in patients in the early stages of the oesophageal adenocarcinoma cascade (gastro-oesophageal reflux disease and Barrett’s oesophagus)^4^. Thus, it would be interesting to determine if some *C. concisus* strains can regulate EMT in oesophageal epithelial cells.

Processes associated with cytoskeletal remodeling and cellular tight junctions were among the most significantly enriched within the upregulated transcripts in IECs infected with *C. concisus* BAA-1457, as well as *C. concisus* ZOT. *C. concisus* strains have been previously shown to modulate the expression of some tight junction proteins^17^, and invade IECs through a paracellular mechanism^10^. Further, it is well established that *Vibrio cholerae* ZOT targets the tight junctions of IECs and induces disassembly^28^. More recently, this was found to occur through a mechanism that involves the activation of proteinase activated receptor 2, PAR2^29^. Importantly, infection with *C. concisus* BAA-1457 and exposure to *C. concisus* ZOT increased the expression of *PAR2* (*F2RL1*) in IECs (1.21- and 1.58-fold, respectively) in this study. Taken together, these findings would suggest that *C. concisus* strains possessing ZOT can target intestinal cellular junctions, as has been observed in other bacterial species possessing this toxin.

In addition to this, purified ZOT was also found to increase the expression of the executioner caspase, *CASP7*. *C. concisus* strains isolated from the oral cavity and faecal samples have previously been shown to induce apoptosis in intestinal epithelial HT-29 cells^10^. Given the role of caspase 7 in apoptosis^30^,^31^, this would suggest that ZOT may promote apoptosis in IECs. This view is supported by upregulation of pro-apoptotic TNF-family pathways and processes associated with death domain receptors and caspases following exposure of IECs to ZOT. Interestingly, the expression of *CXCL16*, a chemokine that promotes TNF-induced apoptosis in macrophages^32^ was found to be increased in IECs both following infection with *C. concisus* BAA-1457 and upon exposure to ZOT. However, the expression of anti-apoptotic cytokines *IL6* and *IL8*, as well as a number of processes related to cell cycle were also significantly increased in IECs exposed to ZOT, suggesting dual activation of competing pathways. Given this, further work is required to clarify the ability and the possible mechanism by which *C. concisus*, and its virulence factor ZOT, may induce cell death. Upregulated transcripts such as *CXCL16* and those within the identified pro-apoptotic pathways may provide insights into this.

This work is not without limitations. Give the costs associated with human RNAseq, global gene expression profiles were analysed using RNAseq for only two *C. concisus* strains. Future studies examining the epithelial responses to a range of clinical and commensal isolates across different time-points would help advance our understanding of the host response to *C. concisus*. While confirmation of gene expression was performed in an additional intestinal epithelial cell-line (HT-29), it would also be beneficial to examine the global response of HT-29 cells, other intestinal epithelial cell-lines (e.g. mucus-producing LS174T cells), and primary intestinal epithelial cells using RNAseq. Further, different RNAseq technologies were employed to examine the epithelial responses to *C. concisus* BAA-1457 and ZOT. While the consistency in the responses suggests good concordance across technologies, it does not rule out the possibility that this may influence assessment of gene expression, particularly for non-coding RNAs.

In conclusion, we comprehensively examined the response of the intestinal epithelial cell-line Caco-2 to *C. concisus*, observing major differences between strains possessing distinct virulence mechanisms. A range of important molecules, pathways and processes that were significantly modulated by this bacterium were identified, thus, providing insights into mechanisms by which *C. concisus* may cause disease. Of particular interest is the ability of *C. concisus* to affect processes related to epithelial-to-mesenchymal transition (NOTCH and Hedgehog signaling), given that this bacterium has been suggested to play a role in Barrett’s oesophagus^4^. We also comprehensively studied the response of the intestinal epithelial cell-line Caco-2 to purified *C. concisus* ZOT, providing evidence that this toxin modulates processes related to cytoskeletal remodeling and cellular tight junctions. This information will serve as a strong basis for future studies addressing the effect of *C. concisus* on epithelial cells, and the role of this bacterium in gastrointestinal disease.

## Methods

### Bacterial culture

*Camyplobacter concisus* strains BAA-1457, UNSWCD, ATCC 51561 and UNSW3 were cultured on Horse Blood Agar (HBA), comprising Blood Agar Base 2 (Oxoid) supplemented with 7% defibrinated horse blood (Oxoid), and incubated for 24–48 h at 37 °C under a microaerobic environment enriched with H_2_. This atmospheric condition was generated using CampyGen and CO_2_Gen gas generating kits (Oxoid) and 0.2 M of sodium borohydride (Sigma Aldrich) dissolved in 10 ml water.

### Expression of Zonula Occludens Toxin from *C. concisus* BAA-1457

ZOT was expressed following the procedures outlined for the PURExpress *In Vitro* Protein Synthesis kit (NEB #E6800; New England Biolabs). Briefly, *C. concisus* BAA-1457 DNA was extracted using the Puregene Core Kit A (Qiagen) according to the manufacturer’s instructions. A linear DNA template containing a T7 promoter sequence, ribosomal binding site (RBS), and the *zot* gene sequence was amplified using a two-step PCR protocol. The first PCR generated a template containing the RBS and *zot* gene using the F1-zot (5′-TAACTTTAAGAAGGAGATATACCAATGCTTAGTTTGATTATCGGTCC) and R1-zot (5′-TATTCACTACTTGTGAGTAGGAAACATAG) primers. This fragment was purified and a second PCR using the primer pair F2-zot (5′-GCGAATTAATACGACTCACTATAGGGCTTAAGTATTAACTTAAGGAGATATACCA) and R2-zot (5′-AAACCCCTCCGTTTAGAGAGGGGTTATATTCACTACTTGTGAGTAGGAA) was performed to generate the final template containing the T7 promoter site before the RBS site and a loop structure following the stop codon. The template was purified and employed to express the ZOT protein using the PURExpress *In Vitro* Protein Synthesis kit according to the manufacturer’s instructions. ZOT was purified using a reverse His-tag purification protocol as outlined by the manufacturer. The purified protein was run on a SDS-PAGE gel, excised from the gel, trypsin digested, and analysed on an Orbitrap Velos ETD (Thermo Electron) mass spectrometer as previously described^19^.

### Mammalian cell culture and infection

Caco-2 cells (American Type Culture Collection: HTB-37) were passaged in Minimal Essential Media (MEM) (Life Technologies), supplemented with 10% v/v Fetal Bovine Serum (FBS) (Bovogen), 1 mM sodium pyruvate, 0.1 mM non-essential amino acids, 2.25 mg l^−1^ sodium bicarbonate and 100 μg ml^−1^ penicillin and streptomycin (Life Technologies), and incubated at 37 °C, with 5% CO_2_. Cell culture medium was replaced every 3–4 days, and cells were passaged approximately every week. HT-29 cells were passaged in McCoy’s 5A Medium Modified (Life Technologies), supplemented with 10% v/v FBS and 100 μg ml^−1^ penicillin and streptomycin, and incubated at 37 °C, with 5% CO_2_. Cell culture medium was replaced every 2 days, and cells were passaged after approximately 5–7 days.

For bacterial infection, Caco-2 or HT-29 cells in antibiotic-free cell culture media were seeded in a 24-well plate (Thermo Fisher Scientific) at a concentration of 1 × 10^6^ cells ml^−1^ and incubated for 48 h at 37 °C with 5% CO_2_. Cells were then infected in triplicate with either *C. concisus* BAA-1457, UNSWCD or UNSW3 at a Multiplicity of Infection (MOI) of 200, or purified ZOT at a concentration of 30 nM. After a 6 h infection period, infected monolayers were washed once with 1 ml DPBS and then RNA extraction was performed using the Isolate II RNA Extraction Mini Kit (Bioline) according to the manufacturer’s instructions. The concentration and quality of extracted RNA was assessed using a NanoDrop ND-1000 (Thermo Fisher Scientific) and stored at −80 °C.

### Library preparation, RNA sequencing and data analysis

For *C. concisus* UNSWCD and BAA-1457, Illumina TruSeq RNA Sample Prep Kit (version 3) was used according to the manufacturer’s instructions for RNA-Seq sample preparation, with 2 μg of total RNA being used per sample (three biological replicates per condition). The libraries were PCR enriched (15 cycles and insert size range: 80 to 330 bp). Six libraries were multiplexed in each lane, using randomisation to control for bias. Libraries were then sequenced on the HiSeq 2000, at the Ramaciotti Centre for Genomics at the University of New South Wales, using TruSeq v3 SBS reagents to generate 100 bp paired-end reads (UNSWCD: Control: 40842678 ± 1799916; Infected: 36991969 ± 1083140; BAA-1457: Control: 38041062 ± 835723; Infected: 35510220 ± 1124242).

For ZOT, the RNA samples were prepared with Illumina TruSeq Stranded Total RNA Kit (Ribozero) using 1 μg of total RNA as input (three biological replicates per condition). The samples were ribosomal RNA depleted as per the manufacturer’s instructions. The libraries were amplified using 12 cycles of PCR. Six libraries were run on a NextSeq500 75 bp PE High Output run (Control: 88799846 ± 8046210; ZOT: 94967481 ± 3083486 paired-end reads).

Sequencing reads were quality checked, filtered, trimmed, and mapped against the human genome as previously described^19^. BioConductor package EdgeR v2.4.6^33^ was used in the R programming environment to identify genes that were significantly differentially expressed between the conditions, following standard normalisation procedures. Data were analysed for regulated pathways and processes using Metacore (Thomson Reuters), and genome-wide transcriptional regulation was visualised using Circos^34^. All RNAseq raw data has been deposited in NCBI Gene Expression Omnibus (GSE84338).

### Autophagy PCR arrays and qPCR validation

cDNA was synthesised from extracted RNA using the RT^2^ First Strand cDNA synthesis kit (Qiagen) according to the manufacturer’s instructions. The concentration and quality of cDNA was assessed using a NanoDrop ND-1000. The cDNA mastermix was prepared using RT^2^ SYBR Green ROX FAST Mastermix (Qiagen) according to the manufacturer’s instructions and loaded onto a Human Autophagy RT^2^ Profiler^TM^ PCR Array (Catalogue No. PAHS-084ZR). Twenty microlitres of cDNA mastermix was loaded into each well of the array, and the array was then sealed using Rotor-Disc Heat-Sealing Film (Qiagen) and a Rotor Disc Heat Sealer (Qiagen). The qPCR was performed using a Rotor Gene Q PCR cycler (Qiagen) under the following cycling conditions: 95 °C for 10 min, 40 cycles of 95 °C for 10 s and 60 °C for 30 s (with acquisition of data occurring during this step), followed by melting curve analysis to verify PCR specificity. The C_T_ threshold was determined manually according to the manufacturer’s recommendations. Data were analysed using online analysis software provided by the manufacturer (Qiagen) using the 2^−ΔΔCT^ method following normalisation against housekeeping gene(s) (Actin beta, *ACTB*; Beta-2-microglobulin, *B2M*; Glyceraldehyde-3-phosphate dehydrogenase, *GADPH*; Hypoxanthine phosphoribosyltransferase 1, HPRT1; Ribosomal protein large P0, *RPLP0*). Quality controls were included within the PCR array to test for reproducibility, reverse transcriptase efficiency and human genomic DNA contamination. Three biological replicates were analysed per condition. Methods for additional qPCR validation of RNAseq data are available in Supplementary Data 3.

## Additional Information

**How to cite this article**: Deshpande, N. P. *et al*. *Campylobacter concisus* pathotypes induce distinct global responses in intestinal epithelial cells. *Sci. Rep.* **6**, 34288; doi: 10.1038/srep34288 (2016).

## Supplementary Material

Supplementary Dataset 1

Supplementary Dataset 2

Supplementary Dataset 3

## Figures and Tables

**Table 1:** Genes within the autophagy pathway differentially expressed following *C. concisus* UNSW3 or BAA-1457 infection of intestinal epithelial cells.

Strain	Gene	Gene Description	Fold Change	Fold Regulation	*P*-value
**UNSW3**	***ATG4A***	ATG4 autophagy related 4 homolog A (*S. cerevisiae*)	0.64	−1.57	0.040
	***HDAC1***	Histone deacetylase 1	0.66	−1.51	0.035
	***HSPA8***	Heat shock 70 kDa protein 8	0.45	−2.21	0.020
	***MAPK14***	Mitogen-activated protein kinase 14	0.60	−1.67	0.0037
	***PIK3C3***	Phosphoinositide-3-kinase, class 3	0.65	−1.54	0.054
	***UVRAG***	UV radiation resistance associated gene	0.73	−1.36	0.077
**BAA-1457**	***ATG4A***	ATG4 autophagy related 4 homolog A (*S. cerevisiae*)	0.47	−2.12	0.0048
	***EIF4G1***	Eukaryotic translation initiation factor 4 gamma, 1	0.37	−2.70	0.021
	***MAP1LC3B***	Microtubule-associated protein 1 light chain 3 beta	2.63	2.63	0.0052
	***MTOR***	Mechanistic target of rapamycin (serine/threonine kinase)	0.52	−1.94	0.0021

Expression was determined using an autophagy-specific qPCR array.

Fold change values  < 1, fold regulation refers to the negative inverse of the fold change. When fold change is ≥1, fold regulation = fold change.

**Table 2:** Regulation of important transcripts associated with pattern recognition receptor signaling pathways upon infection with *Campylobacter concisus* BAA-1457.

Type	ENSEMBL ID	Gene	Fold regulation	*P*-value	Adjusted *P*-value
ALR	ENSG00000163565	*IFI16*	1.23	0.015	0.036
CLR	ENSG00000069493	*CLEC2D*	1.27	0.0011	0.0038
	ENSG00000198178	*CLEC4C*	1.45	1.08E-13	2.09E-12
	ENSG00000152672	*CLEC4F*	1.32	0.017	0.040
NLR	ENSG00000022556	*NLRP2*	1.11	0.0022	0.0070
	ENSG00000167634	*NLRP7*	1.21	5.05E-08	4.44E-07
	ENSG00000185792	*NLRP9*	2.28	7.79E-11	1.05E-09
SLR	ENSG00000161011	*SQSTM1*	1.17	8.24E-06	4.73E-05
	ENSG00000188554	*NBR1*	1.20	1.56E-07	1.26E-06
	ENSG00000123240	*OPTN*	1.18	9.07E-06	5.16E-05
TLR	ENSG00000164342	*TLR3*	1.55	2.53E-07	1.97E-06
	ENSG00000196664	*TLR7*	0.72	0.00033	0.0013
Caspases	ENSG00000106144	*CASP2*	0.89	0.0055	0.015
	ENSG00000164305	*CASP3*	0.77	1.29E-11	1.90E-10
	ENSG00000196954	*CASP4*	1.16	0.00026	0.0011
	ENSG00000132906	*CASP9*	1.29	7.47E-07	5.28E-06
Chemokines	ENSG00000151882	*CCL28*	2.03	8.02E-44	1.17E-41
	ENSG00000107562	*CXCL12*	0.84	2.48E-06	1.59E-05
	ENSG00000145824	*CXCL14*	0.69	1.02E-17	3.00E-16
	ENSG00000161921	*CXCL16*	1.42	1.86E-09	2.06E-08
Cytokines	ENSG00000136244	*IL6*	1.20	0.010	0.026
	ENSG00000145839	*IL9*	0.76	0.0011	0.0038
	ENSG00000150782	*IL18*	1.40	5.10E-09	5.22E-08
	ENSG00000157368	*IL34*	1.41	0.017	0.041
IRF	ENSG00000125347	*IRF1*	1.16	0.00013	0.00056
	ENSG00000168310	*IRF2*	0.83	0.0073	0.020
	ENSG00000126456	*IRF3*	0.90	0.0077	0.021
	ENSG00000117595	*IRF6*	1.30	6.88E-13	1.20E-11

Expression was determined using RNA-seq technology. Results obtained from the EdgeR tool were analysed. A complete list of regulated genes involved in these pathways including cytokine receptors and adaptor molecules can be found in Supplementary Data 1.

ALR: AIM2-like receptors; CLR: C-type lectin receptor; NLR: NOD-like receptor; SLR: sequestosome 1/p62-like receptors; TLR: Toll-like receptor; IRF: Interferon regulatory factors.

**Table 3:** Regulation of important transcripts associated with pattern recognition receptor signaling pathways upon exposure to *C. concisus* Zonula occludens toxin.

Type	ENSEMBL ID	Gene	Fold regulation	*P*-value	Adjusted *P*-value
NLR	ENSG00000022556	*NLRP2*	1.22	0.00046	0.012
	ENSG00000179873	*NLRP11*	0.55	0.00059	0.014
TLR	ENSG00000164342	*TLR3*	1.35	0.0028	0.046
Caspases	ENSG00000165806	*CASP7*	1.30	2.46E-05	0.0011
Chemokines	ENSG00000161921	*CXCL16*	1.39	8.79E-05	0.0030
Cytokines	ENSG00000136244	*IL6*	1.68	6.03E-06	0.00033
	ENSG00000169429	*IL8*	1.44	0.00011	0.0037
IRF	ENSG00000125347	*IRF1*	1.20	0.0022	0.039

Expression was determined using RNA-seq technology. Results obtained from the EdgeR tool were analysed. A complete list of regulated genes involved in these pathways including cytokine receptors and adaptor molecules can be found in Supplementary Data 1.

NLR: NOD-like receptor; TLR: Toll-like receptor; IRF: Interferon regulatory factors.
